# Out of hospital cardiac arrest survivors with inconclusive coronary angiogram: impact of cardiovascular magnetic resonance on clinical management and decision-making

**DOI:** 10.1186/1532-429X-18-S1-O62

**Published:** 2016-01-27

**Authors:** Anna Baritussio, Martina Perazzolo Marra, Amardeep Ghosh Dastidar, Jonathan Rodrigues, Alessandro Zorzi, Angela Susana, Alessandra Scatteia, Estefania De Garate, Giulia Mattesi, Julian Strange, Domenico Corrado, Chiara Bucciarelli-Ducci

**Affiliations:** 1 Bristol NIHR Cardiovascular Biomedical Research Unit, Bristol Heart Institute, Bristol, UK; 2 Department of Cardiac, Thoracic and Vascular Sciences, University of Padua, Padua, Italy

## Background

Non-traumatic out of hospital cardiac arrest (OHCA) is the leading cause of death worldwide, acute coronary syndromes accounting for up to 2/3 of cases. Urgent angiography with a view to primary percutaneous coronary intervention (PPCI) is a class IB recommendation according to international guidelines. Diagnosis and management of patients with unobstructed coronaries or unidentified culprit lesion on angiogram is challenging. Cardiac magnetic resonance (CMR) with its superior tissue characterization is a well-established diagnostic technique. We sought to assess the additive role of CMR in diagnosis and management of patients with an inconclusive coronary angiogram and to determine which findings on CMR best predict clinical impact.

## Methods

We retrospectively analysed our database to collect data on consecutive patients surviving non-traumatic OHCA, undergoing urgent coronary angiogram and CMR. We focused the analysis on patients with an inconclusive angiogram, defined as the evidence of unobstructed coronaries or of coronary artery disease (CAD) without a clear culprit lesion. Clinical impact of CMR was defined as a change in diagnosis, as compared to that made on a multi-parametric pre-CMR basis (clinical history, electrocardiogram, trans-thoracic echocardiogram), or a change in management, which could be a change in medication or the performance/avoidance of invasive procedures (repeat angiogram, myocardial revascularization, ICD implantation).

## Results

Out of 157 patients surviving OHCA referred for CMR after urgent angiogram, we identified 104 patients (78% male, mean age 55.4 ± 16.7) with inconclusive angiogram (66%): 67 patients (64%) had unobstructed coronaries and 37 (36%) had CAD with no clear culprit. Diagnosis based on CMR findings was ischemic heart disease in 42 patients (40%), non-ischemic heart disease in 30 (29%), a structurally normal heart was found in 25 patients (24%) and non-specific findings in 7 (7%). Overall, CMR had a clinical impact in 68/104 patients (65%), determining a change in diagnosis in 17% of patients, a change in management in 31% and both a change in diagnosis and management in 17%. CMR led to myocardial revascularization in 18% of patients and to ICD implantation in 16%; based on CMR findings, an invasive procedure was avoided in 17% of patients. In a multivariate model that included clinical and imaging parameters, LGE (p 0.049, 95% CI 0.09-0.99) and segmental regional wall motion abnormality (p 0.018, 95% CI 1.31-18.9) were the strongest independent predictors of CMR clinical impact (Table 1).

**Table 1:** Predictors of clinical impact

	Sig.	Exp (B)	95% CI for EXP (B)	
			Lower	Upper
Age	0.056	1.030	0.999	1.062
Gender	0.759	0.834	0.262	2.654
LVEF	0.857	0.996	0.948	1.045
LViEDV	0.513	1.008	0.985	1.031
LGE	0.049	0.313	0.098	0.994
Segmental RWMA	0.018	4.985	1.312	18.936
Global RWMA	0.612	1.532	0.294	7.976
Impact of STIR	0.142	2.679	0.718	9.999

LVEF, left ventricular ejection fraction; LViEDV, left ventricular indexed end-diastolic volume; LGE, late gadolinium enhancement; RWMA, regional wall motion abnormality; STIR, short-tau inversion recovery

## Conclusions

CMR had an additive clinical impact on diagnosis and management in 65% of patients surviving OHCA with an inconclusive coronary angiogram. LGE and segmental regional wall motion abnormality were the best independent predictors of clinical impact following CMR. CMR should be enclosed in the clinical-diagnostic work-up of this subgroup of OHCA survivors.

